# The International Quarantine Bureau

**Published:** 1903-09

**Authors:** J. M. Lindsley

**Affiliations:** President, 272 Manhattan Avenue, New York


### THE INTERNATIONAL QUARANTINE BUREAU.

BY J. M. LINDSLEY, M.D., PRESIDENT,
272 MANHATTAN AVENUE, NEW YORK.

Before all the quarantine regulations against yellow fever can be based upon the mosquito as the only means of transmitting the disease and not fomites, it must be proven and accepted by scientific experts who have experimented and studied this question. To get an opinion upon this subject the International Quarantine Bureau addresses a letter to leading doctors, containing the following questions:

1st. Do you believe that the mosquito is the only natural means by which yellow fever is conveyed?

2d. If not, what other?

Our Bureau will give answer of those that accept this as a fact, and then those who hold a different view. Our first answers will be those who served in Cuba and made experiments. After giving the affirmative, we will then take up the negative side of the question.

Dr. W. C. Gorgas, Assistant Surgeon-General, United States Army, and who had charge of sanitary work in Havana, 1898 to 1902, New York City, May 27, 1903:

"I believe that the Stegomyia mosquito is the only means in nature by which yellow fever is conveyed from person to person.

"I hold that success of our sanitary work in Havana demonstrates this. During our work of eradicating that disease in 1901, we paid no attention to fomites of any kind; not only let the soiled clothing, etc., go freely to the wash in the case of the sick in the city, but after its disappearance from the city we placed no prohibition upon baggage and household goods coming in from infected suburbs. The non-immunes from these suburbs were not detained but merely inspected daily for six days. We had during the year twelve hundred non-immunes come into the city from infected points, and from these twelve hundred our inspectors picked up twenty-six cases of yellow fever. The conditions at Havana were about as unfavorable as could exist.

" You will find an interesting discussion in the report of the Seventeenth Annual Meeting of the Conference of State and Provincial Boards of Health, which met last year at New Haven. A committee had been appointed the preceding year to report as to whether the disinfection of baggage was necessary in yellow fever. The report of this committee was that such disinfection was not necessary. I wish you success as it means a great deal in dollars and cents to the business interest of our Southern States."

Dr. V. Havard, Medical Department, U. S. A., West Point, N. Y.:

" In reply to the above would say that I believe the mosquito to be the only natural means by which yellow fever is conveyed. Any one who has read the accounts of our experiments in Havana and still believes in the power of fomites to produce yellow fever must be deaf, dumb and blind."

Dr. James Carroll, Medical Department, U. S. A., Washington, May 16, 1903:

" I am thoroughly and firmly convinced that natural yellow fever is conveyed in no other way than through the bite of the mosquito. The transmission by that agent was first proved in my own person, August 31, 1900, and this observation was subsequently confirmed by ourselves and by Guiteras. Our attempts to infect, by means of fomites all resulted in failure, and the same is true of the attempts made by Havard, Gorgas and Ross in the late summer of 1901.

" The agency of the mosquito explains all the hitherto unexplainable facts in connection with the erratic course of the disease and its non-communicability in certain localities, as well as the interval of time that must elapse between the appearance of the first and the occurrence of subsequent cases. This interval was seldom observed, because the earliest cases of the disease have nearly always been called "bilious remittent fever," "dengue," or something else, until cases occurred with black vomit. Of twenty-two consecutive cases of experimental yellow fever not one had black vomit, though some of them were severe. Guiteras lost three out of eight experimental cases with black vomit, so there is no room for doubt as to the genuine nature of the disease."

Dr. H. R. Carter, Surgeon Public Health and Marine Hospital Service:

1. "I believe that the mosquito Stegomyia Fasciata is the only natural means of conveying yellow fever to man.

2. "That this mosquito is infected in nature only by feeding on one sick of yellow fever."

Baltimore, Md., May 22, 1903.

Dr. R. D. Murrey, Surgeon P. H., and M. H. S.:

Answer to first question—Yes.

Answer to second question—None.

Key West, Florida, May 23, 1903.

Dr. John W. Ross, Medical Director, United States Navy:

"I do believe that the only way in nature for man to contract yellow fever is from the mosquito (Stegomyia), or, in other words, that the mosquito is the only natural means by which yellow fever is conveyed to men seems absolutely demonstrated."

Washington, D. C., June 6, 1903.

---

DR. GARNAULT'S experiments on himself to refute Koch's assertion in regard to the non-identity of human and bovine tuberculosis have resulted negatively, as we have previously reported. No general infection has followed, and the local tubercles on the arm soon healed. No tubercle bacilli nor caseous tissue were found in them. Garnault is an athlete, and possibly the experiments might have resulted differently on a less perfect physique; but as it is, our German exchanges either ignore his experiment altogether, or refer to it as resulting in a confirmation of Koch's statements, instead of a refutation.—*Journal of the American Medical Association.*

